# Molecular Characterization and Functional Analysis of Three Pathogenesis-Related Cytochrome P450 Genes from *Bursaphelenchus xylophilus* (Tylenchida: Aphelenchoidoidea)

**DOI:** 10.3390/ijms16035216

**Published:** 2015-03-06

**Authors:** Xiao-Lu Xu, Xiao-Qin Wu, Jian-Ren Ye, Lin Huang

**Affiliations:** 1 Co-Innovation Center for Sustainable Forestry in Southern China, College of Forestry, Nanjing Forestry University, Nanjing 210037, China; E-Mails: xiaoluxu@163.com (X.-L.X.); jrye@njfu.edu.cn (J.-R.Y.); Lhuang@njfu.edu.cn (L.H.); 2 Jiangsu Key Laboratory for Prevention and Management of Invasive Species, Nanjing Forestry University, Nanjing 210037, China

**Keywords:** pine wood nematode, cytochrome P450 (CYP), rapid-amplification of cDNA ends (RACE), RNA interference (RNAi), detoxification

## Abstract

*Bursaphelenchus xylophilus*, the causal agent of pine wilt disease, causes huge economic losses in pine forests. The high expression of cytochrome P450 genes in *B. xylophilus* during infection in *P. thunbergii* indicated that these genes had a certain relationship with the pathogenic process of *B. xylophilus*. Thus, we attempted to identify the molecular characterization and functions of cytochrome P450 genes in *B. xylophilus*. In this study, full-length cDNA of three cytochrome P450 genes, *BxCYP33C9*, *BxCYP33C4* and *BxCYP33D3* were first cloned from *B. xylophilus* using 3' and 5' RACE PCR amplification. Sequence analysis showed that all of them contained a highly-conserved cytochrome P450 domain. The characteristics of the three putative proteins were analyzed with bioinformatic methods. RNA interference (RNAi) was used to assess the functions of *BxCYP33C9*, *BxCYP33C4* and *BxCYP33D3*. The results revealed that these cytochrome P450 genes were likely to be associated with the vitality, dispersal ability, reproduction, pathogenicity and pesticide metabolism of *B. xylophilus.* This discovery confirmed the molecular characterization and functions of three cytochrome P450 genes from *B. xylophilus* and provided fundamental information in elucidating the molecular interaction mechanism between *B. xylophilus* and its host plant.

## 1. Introduction

As the causal agent of pine wilt disease (PWD), the pine wood nematode (PWN), *Bursaphelenchus xylophilus* (Steiner & Buhrer) Nickle causes huge economic losses by devastating the pine forest and natural landscape resources in Asia, Europe and America [1,2,3,4,5]. In the 1980s, pine wood nematode was found in the Nanjing region of China for the first time [6]. It has now become the most serious introduced forest pest in China. Therefore, effective management strategies to control *B. xylophilus* are urgently needed. Yet, even though the fundamental biological characteristics of *B. xylophilus*, including biological morphology, life history, vectors, host species and ecology related problems have been clarified, based on classical and modern biological research, *B. xylophilus*’ pathogenic mechanism is still uncertain. In addition, with the development of biotechnology techniques in the recent decade, much process about the molecular pathogenicity of *B. xylophilus* has been made [7]. Among them, a number of studies have focused on the pathogenesis-related genes of *B. xylophilus*, especially the cellulase [8], pectatelyase [9] and hemicelluloses genes which are closely related to the cell wall degradation. However, there are still other important pathogenesis-related genes of *B. xylophilus* which remain to be well characterized.

As one of the most versatile enzymes in nature, cytochrome P450 plays an important role in the metabolism of exogenous and endogenous materials and widely exists in all living organisms including protozoa, bacteria, fungi, plants, animals and humans [10,11]. Cytochrome P450 cannot only synthesize and degrade endogenous chemicals [12] such as hormones, fatty acids and steroids, but also metabolize exogenous compounds such as plant secondary metabolites, mutagen and pesticides [13], thus contributing to numerous functions, including growth, development, nutrition, and xenobiotic detoxification. More and more cytochrome P450 genes are being cloned and identified since the first cytochrome P450 gene was cloned in 1983. Recently, a large number of studies confirmed that cytochrome P450 genes from *Caenorhabditis elegans* [14] and some agricultural destructive insects, such as *Helicover armigera* (Hȕbner), *Locusta migratoria*, *Tribolium castaneum* (Herbst) [15,16,17], were associated with their growth, development, reproduction and xenobiotic detoxification. However there is still no report on cytochrome P450 of *B. xylophilus*, and whether it contributes to its functions, including vitality, dispersal ability, reproduction, pathogenicity and pesticide metabolism. The microarray analysis results [18] showed that the expression levels of three CYP450 genes from *B. xylophilus*, *BxCYP33C9*, *BxCYP33C4* and *BxCYP33D3*, were upregulated 6.2-, 4.4-, 3.2-fold, respectively, during infection with *P. thunbergii* compared to *B. xylophilus* cultured on *B. cinerea*, indicating that these CYP450 genes might have a certain relationship with the pathogenic process of *B. xylophilus*.

Therefore, insights into the characteristics of cytochrome P450 genes and their functions in *B. xylophilus* pathogenic process may be helpful in better understanding the molecular interaction mechanism between *B. xylophilus* and its host pines. Since only partial sequences of *BxCYP33C9*, *BxCYP33C4* and *BxCYP33D3* were obtained from DNA microarrays results [18] and the full-length sequences had not been found after searching in the complete genome sequences, we sought to clone the full-length cDNA of the three cytochrome P450s genes using 3' and 5' RACE amplifications. In addition, we also endeavored to silence the three cytochrome P450 genes using RNAi technology in order to assess the functions of the three genes in *B. xylophilus* in our study. These findings not only allow us to understand the role of cytochrome P450 genes in *B. xylophilus*’ pathogenic process, but also provide fundamental information in elucidating the molecular interaction mechanism between *B. xylophilus* and its host plant.

## 2. Results and Discussion

### 2.1. Cloning and Sequence Analysis of Three Cytochrome P450 Genes from B. xylophilus

**Figure 1:**
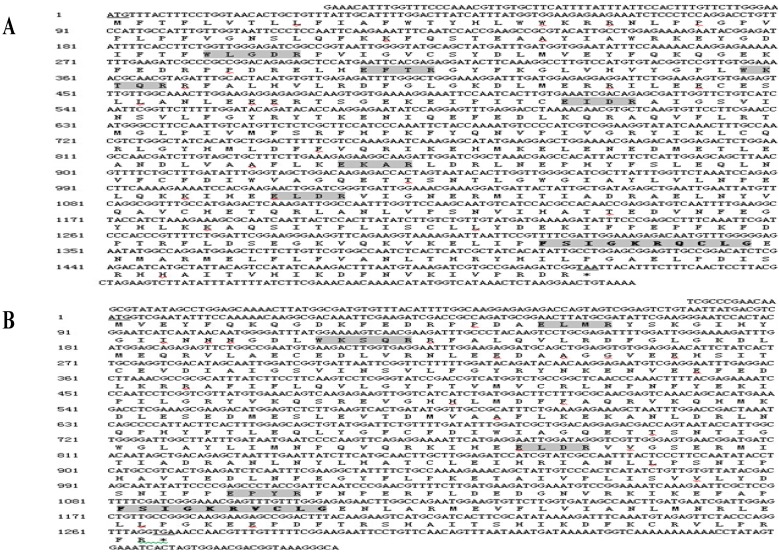

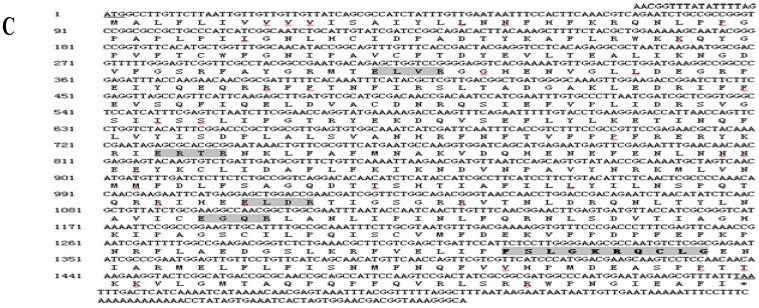
Full-length cDNA sequences and deduced amino acid sequences of BxCYP33C9 (**A**), BxCYP33C4 (**B**) and BxCYP33D3 (**C**) from *B. xylophilus.* Note: The initiation codon and termination codon were underlined. The conserved domains of P450s were shown in dark background. The heme-binding loop region was oblique.

3' RACE and 5' RACE PCR amplification were used to obtain the full-length cDNA sequences of *BxCYP33C9*, *BxCYP33C4* and *BxCYP33D3* from *B. xylophilus*. Sequence analysis showed that the full-length *BxCYP33C9* cDNA was 1663 bp, including a 61-bp 5' untranslated region (UTR), a 96-bp 3' UTR, and a 1506-bp open reading frame (ORF) which encoded for 501 amino acids (Figure 1A). The full-length *BxCYP33C4* cDNA contained 1483 bp, including a 102-bp 5' UTR, a 112-bp 3' UTR, and a 1269-bp ORF which encoded for 422 amino acids (Figure 1B). The full-length cDNA of *BxCYP33D3* was 1688 bp, including a 17-bp 5' UTR, a 141-bp 3' UTR, and a 1530-bp ORF which encoded for 509 amino acids (Figure 1C). Furthermore, conserved domains of the cytochrome P450 family were found in the three deduced amino acid sequences of *B. xylophilus*, such as the typical heme binding loop (FxxGxxxCxG), helix-K (ExxR) and helix-C (WxxxR).

### 2.2. Alignment of the Three Predicted Amino Acid Sequences

**Figure 2:**
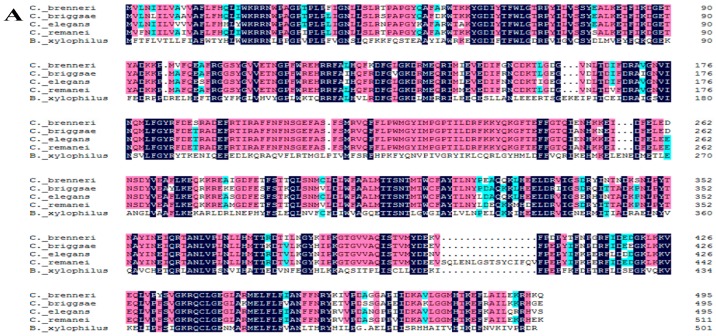

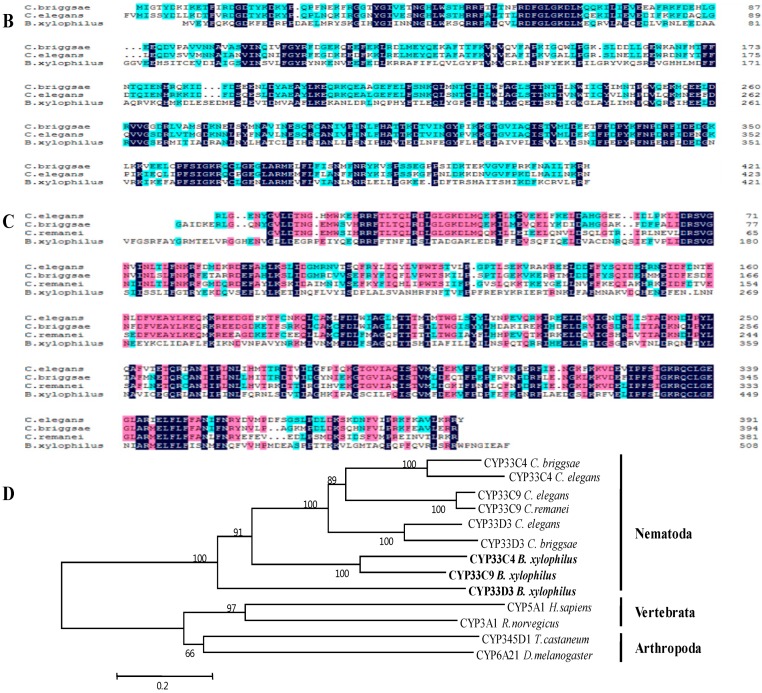
Multiple alignments of predicted BxCYP33C9, BxCYP33C4 and BxCYP33D3 of *B. xylophilus* with other organism CYP proteins. (**A**) Comparison for protein homolo-gogy of CYP33C9 from *B. xylophilus*, *C. brenneri*, *C. elegans*, *C. remanei* and *C. briggsae*; (**B**) Comparison for protein homologogy of CYP33C4 from *B. xylophilus*, *C. elegans* and *C. briggsae*; (**C**) Comparison for protein homologogy of CYP33D3 from *B. xylophilus*, *C. elegans*, *C. remanei* and *C.* *briggsae*; Note: Letters in black boxes, red boxes, and blue boxes indicate invariant amino acid residues, highly conserved amino acid residues, and moderately conserved amino acid residues, respectively, among the CYP proteins; (**D**) Analysis on phylogenetic trees of the deduced CYP33C9, CYP33C4 and CYP33D3 protein sequences with other organism CYP proteins. The full-length CYP proteins sequences utilized in this analysis were as follows: NP_507679.2 (*Caenorhabditis elegans* CYP33D3), XP_002645739.1 (*C. briggsae* CYP33D3), CAP22529.1 (*C. briggsae* CYP33C4), NP_503612.1 (*C. elegans* CYP33C4), NP_503846.1 (*C. elegans* CYP33C9), XP_003115467.1 (*C. remanei* CYP33C9), P24557.3 (*Homo sapiens* CYP5A1), EFA05713. 1 (*Tribolium castaneum* CYP345D1), NP_037237.2 (*Rattus norvegicus* CYP3A1), AAF58186.1 (*Drosophila melanogaster* CYP6A21). KM973210 (*B. xylophilus* CYP33C9), KM973211 (*B. xylophilus* CYP33C4), KM973212 (*B. xylophilus* CYP33D3).

The results of Blastp showed that the deduced amino acid sequence of BxCYP33C9 exhibited a relatively high level of identity with the CYP33C9 protein of *Caenorhabditis brenneri*, *C. briggsae*, *C. elegans* and *C. remanei*, with their sequence percent identity at 42%, 42%, 43% and 41%, respectively. BxCYP33C4 from *B. xylophilus* exhibited a relatively high level of identity with the CYP33C4 protein of *C. elegans* and *C. briggsae*, with their sequence percent identity at 39% and 35%, respectively. Furthermore, the deduced BxCYP33D3 exhibited a relatively high level of identity with the CYP33D3 protein of *C. elegans*, *C. remanei* and *C. briggsae*, with their sequence percent identity at 36%, 36% and 35%, respectively. On this basis, the amino acid sequences which showed relatively high homology with the predicted amino acid sequences were downloaded from NCBI, and DNAMAN software was used to carry out the multiple sequence alignments (Figure 2A–C). MEGA 6.0 software was used to construct phylogenetic trees (NJ) (Figure 2D).

### 2.3. Characteristics of the Three Predicted Proteins

The characteristics of the putative proteins were analyzed with biological information methods. The protein molecular formula of BxCYP33C9, BxCYP33C4 and BxCYP33D3 were C_2695_H_4146_N_714_O_732_S_19_, C_2205_H_3430_N_598_O_633_S_19_, and C_2676_H_4136_N_714_O_739_S_20_, respectively. Their predicted isoelectric points were 6.65, 5.51, and 7.58, respectively. The molecular masses were predicted to be 58, 49 and 58 kDa, respectively, according to the rule that P450s molecular weight for 46–60 kDa. Relatively high quantities of amino acids of BxCYP33C9 were leucine (Leu) (55, 11.0%), glutamate (Glu) (45, 9.0%), and phenylalanine (Phe) (36, 7.2%). Relatively high quantities of amino acids of BxCYP33C4 were leucine (Leu) (44, 10.4%), glutamate (Glu) (40, 9.5%), and arginine (Arg) (30, 7.1%). Relatively high quantities of amino acids of BxCYP33D3 were leucine (Leu) (50, 9.8%), phenylalanine (Phe) (45, 8.8%), Isoleucine (Ile) (39, 7.7%). Results of SignalP 4.1 software showed that there was no signal peptide sequence, while TMHMM server 2.0 showed that there was a transmembrane region between 1–21 amino acids in BxCYP33D3, illustrating that BxCYP33D3 was a membrane binding protein. Unlike BxCYP33D3, results showed that there was no transmembrane structure or signal peptide sequence in the deduced BxCYP33C9 and BxCYP33C4 sequences, indicating that these two proteins of *B. xylophilus* might locate in the cell matrix or organelles since they are hydrophilic. They are likely to exist in a soluble form in organisms.

### 2.4. Detection of RNAi Efficiency

**Figure 3:**
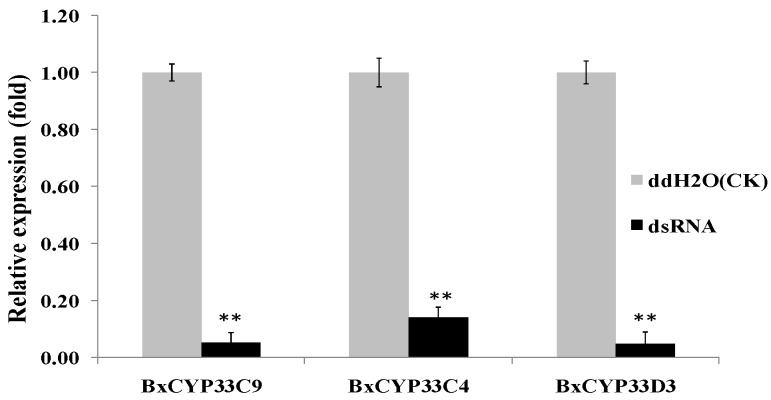
Quantitative real-time RT-PCR analysis of the RNAi efficiency in *B. xylophilus* after treatment with ds*CYP33C9*, ds*CYP33C4* and ds*CYP33D3*. *B. xylophilus* soaked in ddH_2_O was used as control. Data represent mean values ± SD from three independent experiments. Bars show standard deviations of the mean. Asterisks on top of the bars indicate statistically significant differences (** *p* < 0.01, Student’s *t*-test) was found between the dsRNA-treated (ds*CYP33C9*, ds*CYP33C4* and ds*CYP33D3*) and control.

The size of the three dsRNA sequences which contain conservative regions of CYPs, ds*CYP33C9*, ds*CYP33C4* and ds*CYP33D3*, were 641, 428 and 483 bp, respectively. qPCR was used to determine the effect of RNAi on the *BxCYP33C9*, *BxCYP33C4* and *BxCYP33D3* mRNA levels. The *Actin* gene of *B. xylophilus* was utilized as an internal control. Soaking nematodes in ds*CYP33C9*, ds*CYP33C4* and ds*CYP33D3* solution resulted in a significant decrease in *BxCYP33C9*, *BxCYP33C4* and *BxCYP33D3* gene expressions compared to the nematodes soaked in ddH_2_O (control) (Figure 3). Taking the mRNA expression level of the control as 1.00, the mean expression level ofds*CYP33C9*, ds*CYP33C4* and ds*CYP33D3* treated samples were 0.05, 0.14 and 0.05, respectively. These results indicated that *BxCYP33C9*, *BxCYP33C4* and *BxCYP33D3* of *B. xylophilus* had been inhibited by RNAi successfully.

### 2.5. Vitality and Dispersal Ability of B. xylophilus after RNAi

**Figure 4:**
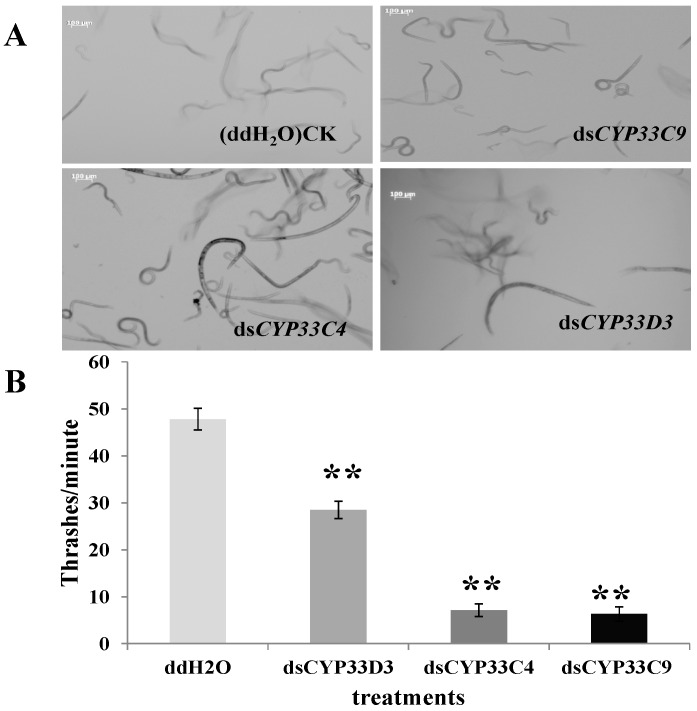
Effect of RNAi to vitality of *B. xylophilus*. (**A**) Morphology and movement of *B. xylophlilus* after soaking in dsRNA (ds*CYP33C9*, ds*CYP33C4* and ds*CYP33D3*) for 48 h. All the pictures were taken under normal conditions (in proper focus). Scale bars = 100 µm; (**B**) Thrashing rate of *B. xylophilus* after soaking in dsRNA. *B. xylophilus* soaked in ddH_2_O were used as control. Data represent mean values ± SD from three independent experiments. Bars show standard deviations of the mean. Asterisks on top of the bars indicate statistical differences (** *p* < 0.01, Student’s *t*-test) were found between the dsRNA-treated (ds*CYP33C9*, ds*CYP33C4* and ds*CYP33D3*) nematodes and controls.

The biological characteristic of *B. xylophilus* after RNAi was demonstrated and analyzed. MostdsRNA-soaked *B. xylophilus* in the Figure 4A were relatively clear compared to the nematodes soaked in ddH_2_O (controls), especially for *B. xylophilus* soaked in ds*CYP33C9* and ds*CYP33C4*, which means they were moving slower than the control. In addition, the thrashing rate of *B. xylophilus* soaked in ddH_2_O, ds*CYP33D3*, ds*CYP33C4*, ds*CYP33C4* were 47, 28, 7, 6 thrashes/min, respectively (Figure 4B). The results indicated that the vitality of PWN was reduced significantly after soaking in dsRNA solutions, especially for *B. xylophilus* soaked in ds*CYP33C9* and ds*CYP33C4*, compared to the nematodes soaked in ddH_2_O. Moreover, results showed that it took a longer time for dsRNA-treated pine wood nematodes to pass through the branch of an equal length of *P. thunbergii* (Table 1), especially for *B. xylophilus* soaked in ds*CYP33C9*. Taken together, these results indicated that some functions related to the vitality and dispersal ability of *B. xylophilus* were likely to be influenced while the genes encoding cytochrome P450 were silenced by RNAi.

**Table 1:** Effect of RNAi to the dispersal ability of *B. xylophilus*.

Treatments	Detected Time (h)	Number of Nematodes
ddH_2_O	12	8.3 ± 2.5
ds*CYP33D3*	24	2.7 ± 1.1
ds*CYP33C4*	96	2.3 ± 1.5
ds*CYP33C9*	144	3.7 ± 1.5

### 2.6. Reproduction and Pathogenicity of B. xylophilus after RNAi

**Figure 5:**
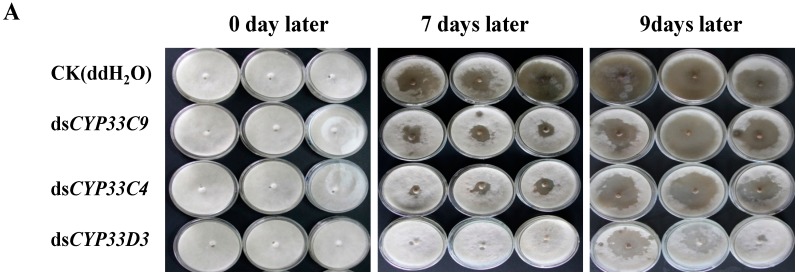

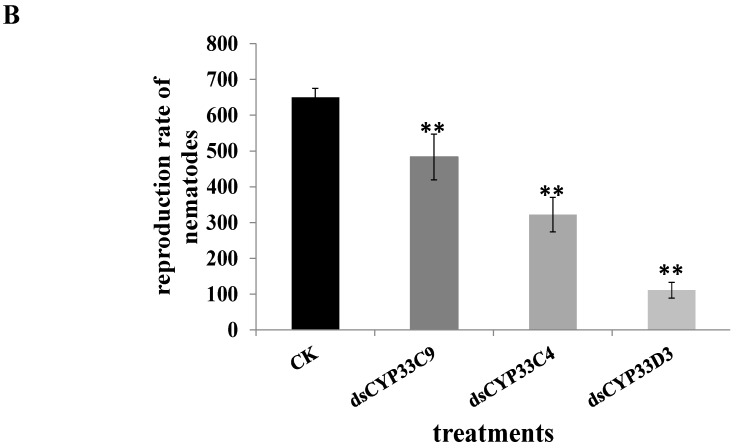
Effect of RNAi on reproduction of *B. xylophilus*. (**A**) RNAi-treated *B. xylophilus* after cultivation with *B. cinerea*; (**B**) Reproduction rate of *B. xylophilus* washed from PDA plate of *B. cinerea* with and without dsRNA (ds*CYP33C9*, ds*CYP33C4* and ds*CYP33D3*) treatment. *B. xylophilus* soaked in ddH_2_O were used as controls. Data represent mean values ± SD from three independent experiments. Bars show standard deviations of the mean. Asterisks on top of the bars indicate statistically significant differences (** *p* < 0.01, Student’s *t*-test) was found between the dsRNA-treated (ds*CYP33C9*, ds*CYP33C4* and ds*CYP33D3*) nematodes and controls.

**Figure 6:**
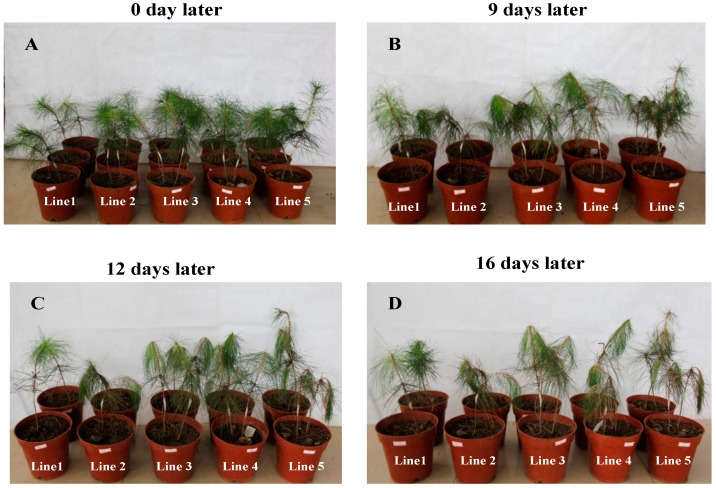
Symptoms in *P. massoniana* seedlings 0 (**A**), 9 (**B**), 12 (**C**) and 16 (**D**) days after inoculation with nematodes soaked in ds*CYP33C9* (line 3), ds*CYP33C4* (line 4) and ds*CYP33D3* (line 5) solution and ddH_2_O (line 2). *P. massoniana* seedlings inoculated with ddH_2_O alone were considered as controls (line 1).

The effect of RNAi on *B. xylophilus* reproduction was performed on PDA plates inoculated with *B. cinerea*. The pine wood nematodes soaked in dsRNA solution showed significantly reduced population compared with the controls (nematodes soaked in ddH_2_O) and this result was confirmed from the *B. xylophilus* feeding situations of different treatments at different periods (Figure 5A). After 9 days at 25 °C, the reproduction rates of nematodes soaked in ds*CYP33D3*, ds*CYP33C4*, ds*CYP33C9* and ddH_2_O were 111-, 322-, 483- and 650-fold, respectively (Figure 5B). These results indicated that feeding and reproduction of *B. xylophilus* were significantly influenced by the RNAi treatment, especially for nematodes with the ds*CYP33D3*-treatment. On the other hand, after the 2-year-old *P. massoniana* seedlings were inoculated with dsRNA-treated *B. xylophilus*, wilting first appeared in the seedlings inoculated with nematodes soaked in ddH_2_O and the wilting rate was 83.3%. Four days later, the seedlings inoculated with nematodes soaked in ds*CYP33D3* solution began to wilt and the wilting rate was 100%. Another four days later, the seedlings inoculated with nematodes soaked in ds*CYP33C4* solution began to wilt and the wilting rate was 83.3%. The seedlings inoculated with nematodes soaked in ds*CYP33C9* solution were the last ones to wilt and the wilting rate was 83.3% (Figure 6). The results indicated that the silencing of *BxCYP33C9*, *BxCYP33C4* and *BxCYP33D3* genes of *B. xylophilus* resulted in a loss of pathogenicity at some level.

### 2.7. Susceptibility of B. xylophilus to Pesticides after RNAi

**Figure 7:**
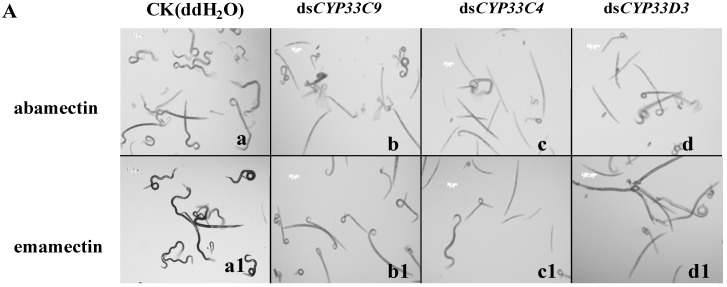

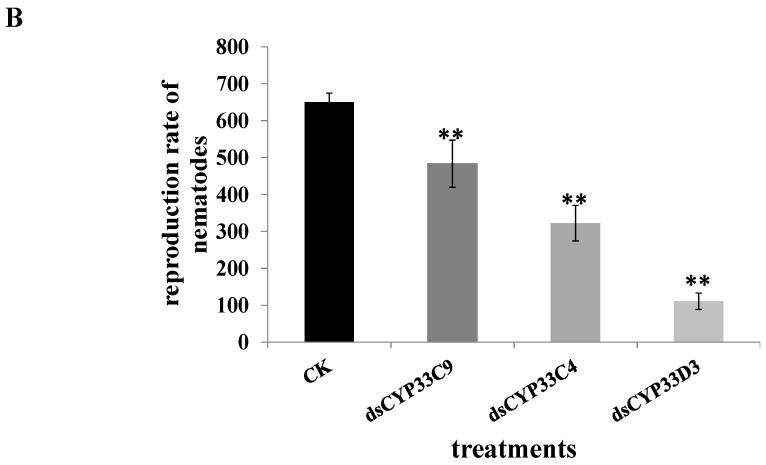
Analysis of susceptibility of *B. xylophilus* to to commonly used pesticides. (**A**) Microscopy analysis of morphology and locomotion of *B. xylophlilus* after soaking in two commonly used pesticides. *B. xylophilus* without RNAi treatment soaked in abamectin (a) and emamectin (a1) solution were conducted as controls. The treatments were ds*CYP33C9*-treated *B. xylophilus* soaked in abamectin (b) and emamectin (b1) solution, ds*CYP33C4*-treated *B. xylophilus* soaked in abamectin (c) and emamectin (c1) solution, dsCYP33D3-treated *B. xylophilus* soaked in abamectin (d) and emamectin (d1) solution. Scale bars = 100 µm; (**B**) Increases in mortalities of *B. xylophilus* with RNAi after soaking in pesticides. *B. xylophilus* without RNAi treatment with a pesticide and those injected with ddH_2_O were considered as controls. Data represent mean values ± SD from three independent experiments. Bars show standard errors of the mean. Asterisks on top of the bars indicate statistical significance (** *p* < 0.01, Student’s *t*-test) of differences between the dsRNA-treated and controls.

To further explore possible insecticide detoxification function of the three cytochrome P450 genes in *B. xylophilus*, nematodes with dsRNA-treatment were soaked in abamectin and emamectin solution, respectively. Observation of *B. xylophilus* under an optical microscope found that the vitality of *B. xylophilus* with the cytochrome P450 dsRNA treatments soaked in pesticides was significantly diminished compared to those with ddH_2_O treatment (Figure 7A). Our results showed that the mortalities of the nematodes treated with ds*CYP33C9*, ds*CYP33C4* and ds*CYP33D3* were 13.7%, 31.5% and 20.5%, respectively, higher than the controls when abamectin was used in our bioassays (Figure 7B). Furthermore, the mortalities of *B. xylophilus* treated with ds*CYP33C9*, ds*CYP33C4* and ds*CYP33D3* were 10.4%, 37.6% and 26%, respectively, higher than the controls when emamectin was used in our bioassays (Figure 7B). These results indicated that the three cytochrome P450 genes may be involved in the resistance of *B. xylophilus* to pesticides.

### 2.8. Discussion

In this article, using RACE technology, we cloned and analyzed the full-length cDNA of three cytochrome P450 genes, *BxCYP33C9*, *BxCYP33C4* and *BxCYP33D3*, from *B. xylophilus*, which had never been reported. Sequence analysis showed that the deduced amino acid sequences of BxCYP33C9, BxCYP33C4 and BxCYP33D3 exhibited a relatively high level of identity with the CYP33C9, CYP33C4 and CYP33D3 proteins of nematodes of *Caenorhabditis*. In addition, RNAi technology was used to demonstrate the functions of the three cytochrome P450 genes. The results indicated that the three genes were associated with the vitality, dispersal ability, reproduction, pathogenicity and pesticide metabolism of *B. xylophilus*. This is the first example of three functional cytochrome P450s from *B. xylophilus*.

To date, there are three hypotheses concerning the pine wood pathogenic mechanism: The enzyme hypothesis, the hollowing-out hypothesis, and the toxins hypothesis [19,20]. Since the plant cell wall is the primary barrier that pine wood nematodes must penetrate [21], cell wall-degrading enzymes such as cellulase [8], pectatelyases [9], and hemicellulose have always been proposed to determine the pathogenicity of pine wood nematodes. Recently, a number of studies have focused on the pathogenesis related genes of *B. xylophilus*, especially the cellulase and pectatelyase genes that are expressed in the esophageal glands of nematodes. Both cellulase and pectatelyase proteins were secreted through the nematode stylet into plant tissues and participated in the weakening of the cell walls, facilitating feeding, penetration and migration of nematodes in pine tissues [8]. Furthermore, many other proteins, such as expansin [22,23], venom-allergen proteins (VAP) [24], heat shock proteins (Hsp) [25,26], and small heat shock proteins (sHSPs) [27,28] were identified to contribute to cell wall degradation, resistance to adverse environment, migration, growth and the development process of *B. xylophilus*. In the present study, three cytochrome P450 genes were cloned from the pine wood nematode and function analysis showed that the silencing of three cytochrome P450 genes reduced dispersal ability of *B. xylophilus* in the host pine trees and its reproduction on *B. cinerea*. These results indicated that besides the cell wall-degrading enzymes, other enzymes might also play important roles in the pathogenic process of *B. xylophilus*. Cytochrome P450, a microsomal mixed function oxidase, plays important roles in metabolizing a wide range of endogenous and exogenous compounds. An important feature of cytochrome P450 enzymes is inducibility, which means that both enzymes’ activity level and gene transcription level of cytochrome P450 were significantly increased when insects or plants were exposed to a pesticide or other exogenous material [29,30]. A series of researches have shown that the presence of plant secondary metabolites and chemical compounds in the environment could induce the expression of CYP450 genes [31,32,33]. *C. elegans* CYP450s have been confirmed to catalyze a series of exogenous and endogenous substrates under different growth conditions [14]. Furthermore, it was proved that the cytochrome P450s of insects, such as *Helicover armigera* (Hȕbner), *Locusta migratoria*, *Tribolium castaneum* (Herbst), play an important role in detoxification of pesticides [15,16,17]. The chemical nature of cytochrome P450-mediated insect resistance is that the cytochrome P450 enzyme system can effectively catalyze the degradation and metabolism of pesticides, which result in the failure of acting on the target site. Our results indicated that this phenomenon may also occur in *B. xylophilus*. In our study, the mortalities of *B. xylophilus* with RNAi treatment were markedly increased when exposed to two kinds of commonly used nematicides, abamectin and emamectin, which indicated that the three cytochrome P450 genes may also play a pesticide detoxification role in *B. xylophilus* and thus improve *B. xylophilus*’ resistance to pesticide. This is the first study of the endogenous substrates of cytochrome P450 in *B. xylophilus*. In addition, in order to combat the invasion by pine wood nematodes, numerous secondary metabolites are generated in pine trees, such as cyclic aromatics and terpenoids [34]. At the same time, *B. xylophilus* must also mobilize reciprocal defensive reactions to avoid damage from the complex compounds. In the present study, the deletion of the *BxCYP33C9*, *BxCYP33C4* and *BxCYP33D3* genes of *B. xylophilus* resulted in a loss of pathogenicity indicating that CYP450s may enhance the pathogenicity role of *B. xylophilus* through the mobilization of *P. massoniana* secondary metabolites or the production of toxic metabolites that damage the pine trees. However, the specific substrates of CYP450s in *B. xylophilus* need to be further investigated.

Overall, this study focused on the molecular characterization and functional analysis by RNAi of three cytochrome P450 genes from *B. xylophilus*. These discoveries would provide a better understanding of the molecular mechanism of pathogenesis of *B. xylophilus* and contribute to management of pine wood nematodes in the future.

## 3. Experimental Section

### 3.1. Materials

The highly virulent AMA3 strain of *B. xylophilus* used in this study was isolated from wood chips of infested *Pinus thunbergii* Parl from Maanshan, Anhui, China, which was maintained in the forest pathology laboratory of Nanjing Forestry University, Nanjing, China. The *Botrytis cinerea* strain was also provided by our laboratory. Two-year-old *P. massoniana* seedings and five-year-old *P. thunbergii* used in this study were grown in the greenhouse (32°077'N, 118°808'E) at 30 °C during the daytime and 25 °C at night, with 70% relative humidity. Two commonly used pesticides, abamectin and emamectin, were purchased from Hebei Veyong Bio-Chemical Pesticide Co., Ltd. (Shijiazhuang, China).

### 3.2. Collection of B. xylophilus

Isolate of *B. xylophilus* was grown in *B. cinerea* culture on potato dextrose agar (PDA) plates at 25 °C for 5–7 days. Baermann funnel method was used to separate *B. xylophilus* from PDA plates. *B. xylophilus* was collected by centrifugation at 3500 rpm for 3 min and washed three times with distilled water. The nematodes were immediately frozen in liquid nitrogen and eventually stored at −80 °C in a 1.5 mL centrifuge tube for subsequent RNA extraction.

### 3.3. RNA Extraction of B. xylophilus

The collected nematodes were ground into powder in a mortar after joining the liquid nitrogen. Total RNA of nematodes was extracted using an optimized Trizol method with Trizol reagent (Invitrogen, Waltham, MA, USA). The RNA was measured by ultraviolet absorbance at A260/280 (Eppendorff AG 22331, Hamburg, Germany) and examined by electrophoresis on a 1% agarose gel.

### 3.4. Full-Length cDNA Cloning of P450s from B. xylophilus

The full-length *BxCYP33C4* and *BxCYP33D3* cDNA were obtained using the 3'-Full RACE Core Set with PrimeScript™ RTase kit (TaKaRa Biotechnology, Dalian, China) and the full-length cDNA of *BxCYP33C9* was obtained using 5'-Full RACE Kit with TAP (TaKaRa Biotechnology, Dalian, China). Gene-specific primers GSP1 (for *BxCYP33C4*) and GSP2 (for *BxCYP33D3*), GSP3 (for *BxCYP33C9* first round of PCR) and GSP4 (for *BxCYP33C9* second round of PCR) (Table 2) were, respectively, designed for 3' and 5' RACE amplification based on three partially known sequences of *BxCYP33C4*, *BxCYP33D3* and *BxCYP33C9* which were obtained from the DNA microarrays results [18]. The amplification profiles were all as follows: A cycle at 94 °C for 3 min, 30 cycles at 94 °C for 30 s, 55 °C for 30 s, 72 °C for 2 min, and 72 °C for10 min. The resulting nested PCR products were purified and recycled according to the Gel Extraction Kit (Axygen, Hangzhou, China) manufacturer’s instructions. Subsequently, the amplified PCR products were cloned into the pEASY-T1 vector (TransGen Biotech, Beijing, China), and transformed into *Escherichia coli* Trans1-T1 competent cells (TransGen Biotech, Beijing, China) and then plated on a selective medium. A single colony of the transformants was inoculated and cultured at 37 °C in LB medium containing ampicillin (50 µg/mL) with shaking for 4 h. The fresh bacterial suspension liquid was sequenced at Nanjing Genscript sequencing company (Nanjing, China) after PCR detection with primers M13F (−47) and M13R (−48) (Table 1).

### 3.5. Bioinformatic Analysis

Amino acid sequences of homologous BxCYP33C9, BxCYP33C4 and BxCYP33D3 proteins from other organisms were obtained from NCBI using Blastp (available online: http://blast.ncbi.nlm. nih.gov/Blast.cgi). Multiple sequence alignment of deduced protein sequences was carried out with DNAMAN software and phylogenetic analysis of these two P450 proteins was carried out with MEGA 6.0 software using the neighbor-joining (NJ) method. The characteristics of predicted BxCYP33C9, BxCYP33C4 and BxCYP33D3 proteins such as amino acids composition prediction and protein molecular weight and isoelectric point calculation (available online: http://cn.expasy.org/tools/protparam.html), signal peptide prediction (available online: http://genome.cbs.dtu.dk/services/SignalP), prediction of transmembrane helices in proteins (available online: http://www.cbs.dtu.dk/services/TMHMM/) were analyzed on the bioinformatics website.

**Table 2:** PCR primers used in the present study.

Name of Primer	Sequence (5'-3')
cDNA Cloning of three cytochrome P450 genes	
3'RACE outer primer	TACCGTCGTTCCACTAGTGATTT
GSP1	TTCGCCCGAACAAGCGTATA
GSP2	AATGTTGGACTGCTGGATGA
5'RACE outer primer	CATGGCTACATGCTGACAGCCTA
GSP3	TGTGATAGCGATGAGTGA
5'RACE inner primer	CGCGGATCCACAGCCTACTGATGATCAGTCGATG
GSP4	TTGAAAGGCTCGGGAAAT
M13F(−47)	CGCCAGGGTTTTCCCAGTCACGAC
M13R(−48)	AGCGGATAACAATTTCACACAGGA
Preparation of template DNA for dsRNA	
*Bx*C4T7F	TAATACGACTCACTATAGGGTTGGGAGTGAACGGATGA
*Bx*C4R	GATCGCATGACTTCTTGTA
*Bx*C4F	TTGGGAGTGAACGGATGA
*Bx*C4T7R	TAATACGACTCACTATAGGGGATCGCATGACTTCTTGTA
*Bx*C9T7F	TAATACGACTCACTATAGGGGCAGCTATGATTGATGGT
*Bx*C9R	CAATGAGAAGTAATGTGGC
*Bx*C9F	GCAGCTATGATTGATGGT
*Bx*C9T7R	TAATACGACTCACTATAGGGCAATGAGAAGTAATGTGGC
*Bx*D3T7F	TAATACGACTCACTATAGGGGCCCGAGATTTACCAAGA
*Bx*D3R	AACGCATCAATCAGACACTT
*Bx*D3F	GCCCGAGATTTACCAAGA
*Bx*D3T7R	TAATACGACTCACTATAGGGAACGCATCAATCAGACACTT
Real time PCR	
qC9F1	GCGGTTTGCCATGAGACT
qC9R1	AAACGGGTGGGATCGAAT
qC4F1	AAGATCGACCGCCAGATG
qC4R1	CACCTCCAGCTGCATCCT
qD3F1	CTGATGGGGCAAAGTTGG
qD3R1	GCGGGTCCGAAATGTAGA
Actin F	GCAACACGGAGTTCGTTGTAGA
Actin R	GTATCGTCACCAACTGGGATGA

The T7 promoter sequences were underlined.

### 3.6. RNA Interference (RNAi)

RNA interference (RNAi) was first described in *Caenorhabditis elegans* for analysis of gene function [35]. Later, RNAi was developed as an effective tool used in plants and animals for gene function study and genetic manipulation [36,37,38]. In this study, RNAi technology was used to assess the functions of the three cytochrome P450 genes in *B. xylophilus*. Recombinant plasmids were extracted from the bacteria liquid which had been shook at 200 rpm for 24 h at 37 °C according to the AxyPrep™ Plasmid Mini prep Kit manufacturer’s protocol (Axygen, Hangzhou, China). The DNA templates used for synthesizing double-stranded RNA (dsRNA) were amplified with the primers in Table 2 and then dsRNA was synthesized using the MEGscript RNAi Kit (Ambion Inc., Austin, TX, USA). The RNAi soaking method used in this study was performed according to a previous report [39]. Approximately 3000 individuals (a mix of juveniles and adults) of freshly cultured *B. xylophilus* were soaked in 50 µL dsRNA (800 ng/µL) after being washed with distilled water for 3 times at 3500 rpm for 3 min and then incubated at 180 rpm for 48 h at 20 °C. The same number of nematodes soaked in 50 µL ddH_2_O was used as the negative control. Each treatment had three replicates. Samples from each treatment were washed thoroughly with ddH_2_O for several times after soaking and then used for additional experiments.

### 3.7. Quantitative Real-Time RT-PCR

In order to verify that *BxCYP33C9*, *BxCYP33C4* and *BxCYP33D3* of *B. xylophilus* had been knocked down by RNAi successfully, quantitative real-time PCR (qPCR) was used to detect interference efficacy. Total RNA extractions from *B. xylophilus* after RNAi were carried out as described previously in our study. The first-strand cDNA was then synthesized using a cDNA synthesis Kit (TransGen Biotech, Beijing, China) with 2 µg of total RNA according to the manufacturer’s instructions. The synthesized cDNA was stored at −20 °C after being measured by ultraviolet absorbance at A260/280 (Eppendorff AG 22331, Hamburg, Germany). qPCR was then carried out using TransStart Green qPCR Super Mix (TransGen Biotech, Beijing, China) and ABI Prism 7500 (Applied Biosystems, Foster City, CA, USA), with the primers listed in Table 2. The thermal profile used was: 94 °C for 30 s, 40 cycles of 94 °C for 5 s, and 60 °C for 34 s. *Actin* gene of *B*. *xylophilus* was utilized as internal control. Relative expression levels were determined using the ABI Prism 7500 software and 2^−ΔΔ*C*t^ method. qPCR was carried out with three biological replicates and three technical replicates.

### 3.8. Analysis of Vitality and Dispersal Ability of B. xylophilus after RNAi

The vitality of *B. xylophilus* was examined and photographed using a Zeiss Axio Image M2 microscope (Zeiss MicroImaging GmbH, Oberkochen, Germany). About 100 nematodes from each treatment were observed. If moving too fast, *B. xylophilus* cannot be photographed clearly, so the sharpness of nematodes in the photographs reflected swing speed of *B. xylophilus* to some extent. In addition, thrashing rate was conducted for evaluating the vitality of *B. xylophilus* according to Tsalik and Hobert [40]. After a 2-min recovery period, thrashes were counted for 1 min. A thrash of *B. xylophilus* was defined as the head from one direction to another direction, and then the same action was repeated again. The detection of *B. xylophilus*’ dispersal ability was performed according to a previous method [41]. First, a rubber tubing (about 3 cm in height, 1 cm in diameter) was upended on one end of a fresh *P. thunbergii* branch (about 5 cm in length, 1 cm in diameter), and the other end of the branch was then put into a small dish with 1 mL sterile water. Then a piece of sterile absorbent cotton was put into the rubber tubing. All of these were put into a bottle in the end. Subsequently, 200 µL of mixed suspension containing 150 nematodes was dropped on the absorbent cotton. The water in the dish was observed with optical microscopy during a 6-h interval and the number of nematodes and the time of its first appearance were recorded during the time. Each treatment in this experiment has three replicates and the experiment was repeated twice.

### 3.9. Analysis of Reproduction and Pathogenicity of B. xylophilus after RNAi

Fifteen pairs of female and male *B. xylophilus* with RNAi treatment were picked and cultured on a PDA plate with *B. cinerea* at 25 °C for 9 days. The feeding situation of *B. xylophilus* was observed and photographed periodically. Subsequently, the nematodes were washed off the plates and counted with an optical microscope. In order to determine the pathogenicity of *B. xylophilus* after RNAi, 1000 nematodes were injected into each 2-year-old *P. massoniana* seedling, according to a previous method [42]. *B. xylophilus* soaked in ddH_2_O without dsRNA and ddH_2_O alone were utilized as controls. Photographs were taken regularly to record the seedlings’ infection state. Each control group in this experiment has six individuals.

### 3.10. Analysis of Susceptibility of B. xylophilus to Pesticides after RNAi

At present, a large number of studies on the cytochrome P450 genes were focused on its detoxification function to pesticides, especially on insects such as *Helicover armigera* (Hȕbner), *Locusta migratoria*, *Tribolium castaneum* (Herbst) [15,16,17]. To further explore possible insecticide detoxification roles of the three cytochrome P450 genes in *B. xylophilus*, two kinds of commonly used nematicides, abamectin and emamectin, were used to conduct on previously dsRNA-soaked nematodes. Thus, 200 nematodes were soaked in 500 µL abamectin (4.5 mg/L) and emamectin (0.3 mg/L) solutions, respectively [43], in a 1.5 mL centrifuge tube. *B. xylophilus* without RNAi-treatment soaked in nematicides and ddH_2_O, were used as controls. The Zeiss Axio Image M2 microscope was used to observe the vitality and morphology of *B. xylophilus*, and photographs were taken after the nematodes were soaked at 25 °C for 24 h.

### 3.11. Statistical Analysis

All assays were performed in triplicate as three independent trials. Results showed as the mean ± standard deviation (SD) of three independent experiments were calculated using Microsoft Excel. The statistical significance was determined using SPSS Statistics 17.0 software (IBM China Company Ltd., Beijing, China) to perform the paired *t*-tests. Asterisks indicate statistically significant differences (* *p* < 0.05, ** *p* < 0.01, Student’s *t*-test).
